# Impact of supine vs. hip- and knee-flexed position on anteroposterior diameter and inclination of the female pelvic outlet: an MRI-based 3D reconstruction study

**DOI:** 10.3389/fmed.2026.1895286

**Published:** 2026-08-21

**Authors:** Mengjie Yan, Xiaobing Yang, Yidan Wang, Mingting Wang, Yaping Wang, Maohong Gu

**Affiliations:** 1 Department of Obstetrics and Gynecology, Nanjing First Hospital, Nanjing Medical University, Nanjing, Jiangsu, China; 2 Department of Pathology, Nanjing First Hospital, Nanjing Medical University, Nanjing, Jiangsu, China; 3 Department of Ultrasound, Nanjing First Hospital, Nanjing Medical University, Nanjing, Jiangsu, China

**Keywords:** hip knee flexion, magnetic resonance imaging, maternal position, pelvic morphology, three-dimensional reconstruction

## Abstract

**Background:**

Maternal positioning influences pelvic biomechanics during labor; however, the anatomical mechanisms underlying position-related pelvic morphological changes remain incompletely understood. This study aimed to quantitatively evaluate pelvic morphological changes associated with a hip- and knee-flexed position compared with the supine position using three-dimensional (3D) reconstruction.

**Methods:**

In total, 27 nulliparous non-pregnant women underwent pelvic MRI examinations in both the supine and hip- and knee-flexed positions. A radiation-free MRI-based 3D reconstruction approach was used to measure pelvic diameters and angles. A total of 12 pelvic parameters were evaluated, including the pelvic inlet, midpelvis, outlet, external pelvic measurements, and pelvic inclination. Data normality was assessed before paired-samples t-tests were performed, and intraclass correlation coefficients were calculated to assess measurement reliability.

**Results:**

Compared with the supine position, the hip- and knee-flexed position significantly increased the anteroposterior diameter of the pelvic outlet (mean increase, 4 mm; *p* = 0.0361) and significantly decreased pelvic inclination (mean reduction, 25°; *p* < 0.001). No significant differences were observed in the pelvic inlet, midpelvic, or other external pelvic measurements (*p* > 0.05). All measurements demonstrated excellent inter-rater reliability, with intraclass correlation coefficients exceeding 0.90.

**Conclusion:**

A hip- and knee-flexed position is associated with specific and quantifiable changes in pelvic outlet morphology and pelvic orientation in non-pregnant women. These findings provide preliminary anatomical and biomechanical evidence regarding position-related pelvic mobility. Further studies in pregnant populations are required to determine the clinical relevance of these positional changes during labor.

## 1 Introduction

Maternal positioning may influence the mechanics of labor by altering pelvic orientation and the spatial relationship between the maternal pelvis and the fetus. However, clinical evidence regarding the benefits and risks of individual positions remains heterogeneous. Systematic reviews evaluating upright, squatting, hands-and-knees, and thigh-flexion positions have reported variable effects on labor duration, operative birth, and other maternal or neonatal outcomes, with findings influenced by the stage of labor, epidural analgesia, the specific position adopted, and the outcome assessed (1–4). Quantitative imaging and biomechanical modeling are therefore important for clarifying the anatomical changes associated with maternal repositioning.

Previous imaging and modeling studies have demonstrated that pelvic dimensions are position-dependent. Using open magnetic resonance imaging (MRI) in non-pregnant women, Michel et al. (5) found that the hands-and-knees and squatting positions increased selected midpelvic and outlet dimensions compared with the supine position. Reitter et al. (6) subsequently used MRI to compare the supine and kneeling squat positions in pregnant and non-pregnant women and reported increases in selected anteroposterior and transverse dimensions of the midpelvis and pelvic outlet in the kneeling squat position. More recently, Kjeldsen et al. (7) examined 50 pregnant women in the supine, semi-lithotomy, and kneeling squat positions. At gestational week 32, the mean pelvic outlet diameter was 0.2 cm larger in the semi-lithotomy position than in the supine position, whereas shifting from the supine to the kneeling squat position increased selected midpelvic and outlet dimensions by up to approximately 1 cm. Computational modeling by Hemmerich et al. (8) similarly showed that, under pregnant simulation conditions, the final squat posture increased the anteroposterior and transverse pelvic outlet diameters by 6.1 and 11.0 mm, respectively. Nevertheless, a systematic review of late-pregnancy MRI studies found that the currently available MRI markers did not reliably predict labor outcomes (9). Thus, imaging and modeling studies demonstrate that pelvic dimensions are position-dependent; however, the clinical significance of these anatomical changes remains uncertain.

Contemporary pelvic assessment incorporates CT-, ultrasound-, and MRI-based approaches. Recent publications have addressed population-specific CT pelvimetry reference values, intrapartum ultrasound, external pelvimetry, automated three-dimensional CT measurement, population-specific 3D CT pelvimetry, and the application of 3D pelvimetry to pelvic surgical planning (10–15). MRI-based methods have also been applied to the evaluation and reconstruction of pelvic floor muscles and pelvic organ support structures, as well as to the assessment of pelvic surgical difficulty and obstetric outcome prediction (16–21). A direct comparison of 3D pelvic models derived from CT and MRI demonstrated good agreement and reproducibility, supporting the technical feasibility of MRI-based 3D pelvimetry (22). However, randomized evidence has not established sufficient benefit for routine X-ray pelvimetry in determining the mode of delivery in women with cephalic presentations (23), and conventional MRI remains limited in depicting cortical bone because of its very short apparent transverse relaxation times (24).

The McRoberts maneuver is commonly recommended as an initial external maneuver in the emergency management of shoulder dystocia rather than as a routine maternal birthing position (25). Recent clinical research has primarily evaluated the frequency, success, and maternal or neonatal complications associated with different shoulder dystocia maneuvers rather than quantitatively assessing pelvic morphology during the McRoberts maneuver (26). Moreover, its anatomical mechanism remains incompletely understood (25). A biomechanical study demonstrated that, regardless of the initial degree of thigh abduction, the McRoberts maneuver resulted in elevation of the pubic symphysis and reduction of lumbar lordosis (27). Earlier X-ray pelvimetry also demonstrated cephalad rotation of the pubic symphysis and changes in pelvic and lumbosacral orientation during the maneuver (28). However, these studies used biomechanical or radiographic methods rather than MRI-based three-dimensional reconstruction and did not comprehensively quantify positional changes in the pelvic inlet, midpelvis, outlet, sacral parameters, and pelvic inclination. Therefore, comprehensive MRI-based three-dimensional evidence regarding pelvic dimensional changes in a posture approximating the principal hip-flexion component of the McRoberts maneuver remains limited.

In the present study, the term “hip- and knee-flexed position” refers to an MRI-compatible posture in which both hips and knees are flexed, and both thighs are drawn toward the abdomen within the spatial constraints of the MRI scanner. This position was designed to approximate the principal hip-flexion component of the McRoberts maneuver but should not be regarded as an exact reproduction of the complete clinical maneuver. Using radiation-free MRI-based three-dimensional reconstruction, this study aimed to quantitatively compare pelvic diameters and angles between the supine and hip- and knee-flexed positions in nulliparous non-pregnant women. A secondary exploratory objective was to describe the distribution of pelvic morphological types in the study sample.

## 2 Materials and methods

### 2.1 Study design

This study was a prospective, self-controlled (paired-design) comparative study.

### 2.2 Ethical approval

This study was approved by the Ethics Committee of Nanjing First Hospital (Approval No: KY20260518-KS-01). All participants provided written informed consent before undergoing the examination. The study complied with the ethical principles of the Declaration of Helsinki.

### 2.3 Study participants

A total of 27 Asian women of childbearing age who volunteered to undergo pelvic MRI examination at Nanjing First Hospital were enrolled.

The inclusion criteria were as follows: (1) age 21–35 years; (2) body mass index (BMI) 18–24.9 kg/m^2^; (3) height 155–165 cm; (4) weight 45–65 kg; (5) nulliparous and non-pregnant. The restricted height, weight, and BMI ranges were selected to reduce variability in body habitus and to facilitate the evaluation of within-participant positional changes in this small paired study.

The exclusion criteria were as follows: (1) contraindications to MRI; (2) congenital pelvic malformation; (3) history of trauma or surgery involving the pelvis, spine, or lower limbs; (4) history of pelvic surgery; and (5) history of delivery or induced abortion.

Menstrual-cycle phase, reproductive hormone concentrations, and hormonal contraceptive use were not prospectively recorded.

The basic demographic characteristics of the 27 participants are presented in Table 1. The data indicate a high degree of homogeneity within the sample.

**Table 1:** Basic demographic characteristics of the 27 study participants.

Index	Mean ± SD	Range
Age (years)	29.5 ± 1.91	26–32
Height (cm)	161.1 ± 2.97	155–165
Weight (kg)	56.95 ± 3.72	48–62
BMI (kg/m^2^)	21.94 ± 1.31	18.75–24.22

### 2.4 Equipment and software

The MRI examinations were performed using a Philips Ingenia 3.0-T wide-bore MRI scanner with a 70-cm bore diameter (Philips Healthcare, Best, the Netherlands). Three-dimensional reconstruction and statistical analyses were performed using Mimics 17.0 software (Materialise, Leuven, Belgium) and SPSS 22.0 software (IBM Corp., Armonk, NY, USA), respectively.

### 2.5 Study procedures

#### 2.5.1 Scanning positions

All participants underwent MRI examinations in both the supine and hip- and knee-flexed positions using the same 70-cm wide-bore scanner.

#### 2.5.2 Supine position

The participant lay supine in the center of the examination table, with the midsagittal plane perpendicular to the table surface, both hands placed behind the head, and both lower limbs extended and adducted.

#### 2.5.3 Hip- and knee-flexed position

The participant flexed both hips and knees, placed both hands in the popliteal fossae, and drew both thighs toward the abdomen as far as possible within the available bore space. The participant maintained this position during image acquisition while the pelvis remained within the predefined scanning range.

The hip- and knee-flexed position was intended to approximate the principal hip-flexion component of the McRoberts maneuver within the spatial constraints of the MRI scanner rather than to reproduce every component of the complete clinical maneuver. The two scanning positions are illustrated in Supplementary Figure 2.

#### 2.5.4 Scanning sequence and parameters

The same imaging sequence, scan range, and acquisition parameters were used for both scanning positions.

A non-fat-saturated fast spin echo T2-weighted imaging (FSE-T2WI) axial thin-slice sequence was used. The optimized scanning parameters were as follows: repetition time (TR)/echo time (TE) = 3000/102 ms; flip angle = 180°; field of view (FOV) = 360 mm × 360 mm; number of excitations (NEX) = 3; slice thickness/interslice gap = 3.0 mm/0 mm; matrix = 512 × 512; voxel size = 0.5 mm × 0.5 mm × 3.0 mm. The scanning range extended from the upper margin of the fifth lumbar vertebra to the lower margin of the ischial tuberosities.

#### 2.5.5 Three-dimensional model reconstruction

**Figure 1:**
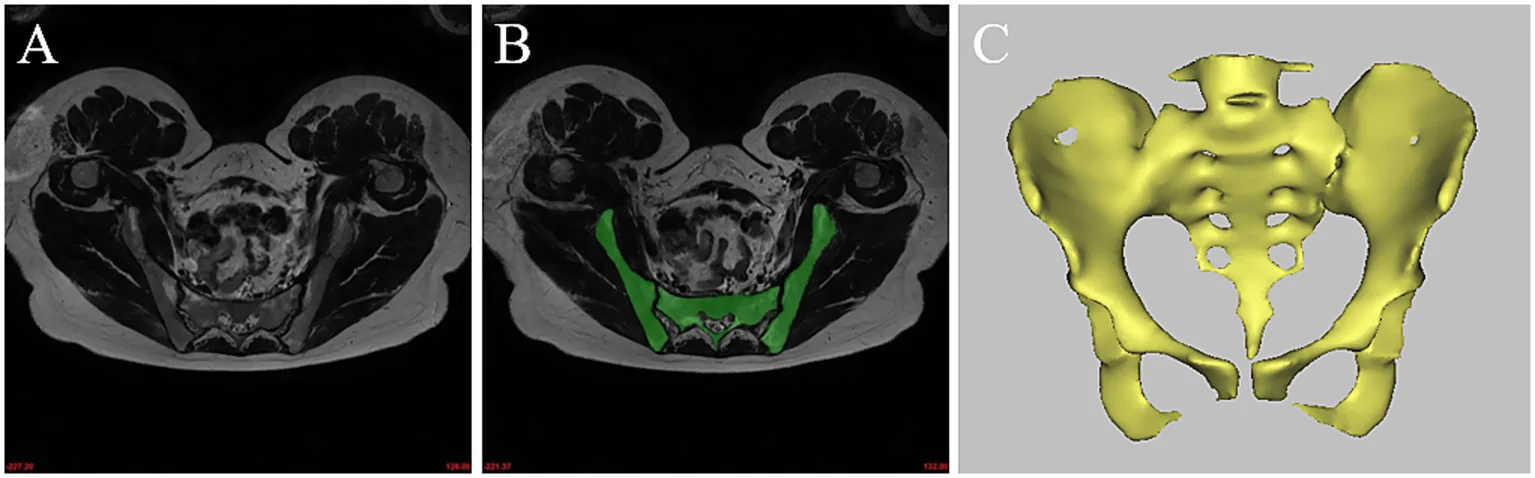
Methodological flowchart of MRI three-dimensional reconstruction. **(A)** Representative axial FSE-T2WI image. **(B)** Bone tissue mask generated using threshold segmentation and mask boundary correction. **(C)** Final three-dimensional pelvic model generated after region growing, three-dimensional reconstruction, and smoothing.

The original MRI data in Digital Imaging and Communications in Medicine (DICOM) format were imported into Mimics 17.0. The three-dimensional reconstruction workflow is shown in Figure 1: (A) selection of representative axial FSE-T2WI images; (B) generation of bone tissue masks using threshold segmentation and mask boundary correction; and (C) region growing, three-dimensional mask reconstruction, and smoothing to generate a digital three-dimensional model of the female pelvis, including part of the lumbar spine.

### 2.6 Measured parameters and measurement methods

Pelvic measurements were performed on the MRI-based three-dimensional pelvic models using the measurement tools in Mimics 17.0. Each reconstructed model was rotated to the anatomical view required to identify the corresponding bony landmarks. Linear measurements were recorded in millimeters, and angular measurements were recorded in degrees.

**Figure 2:**
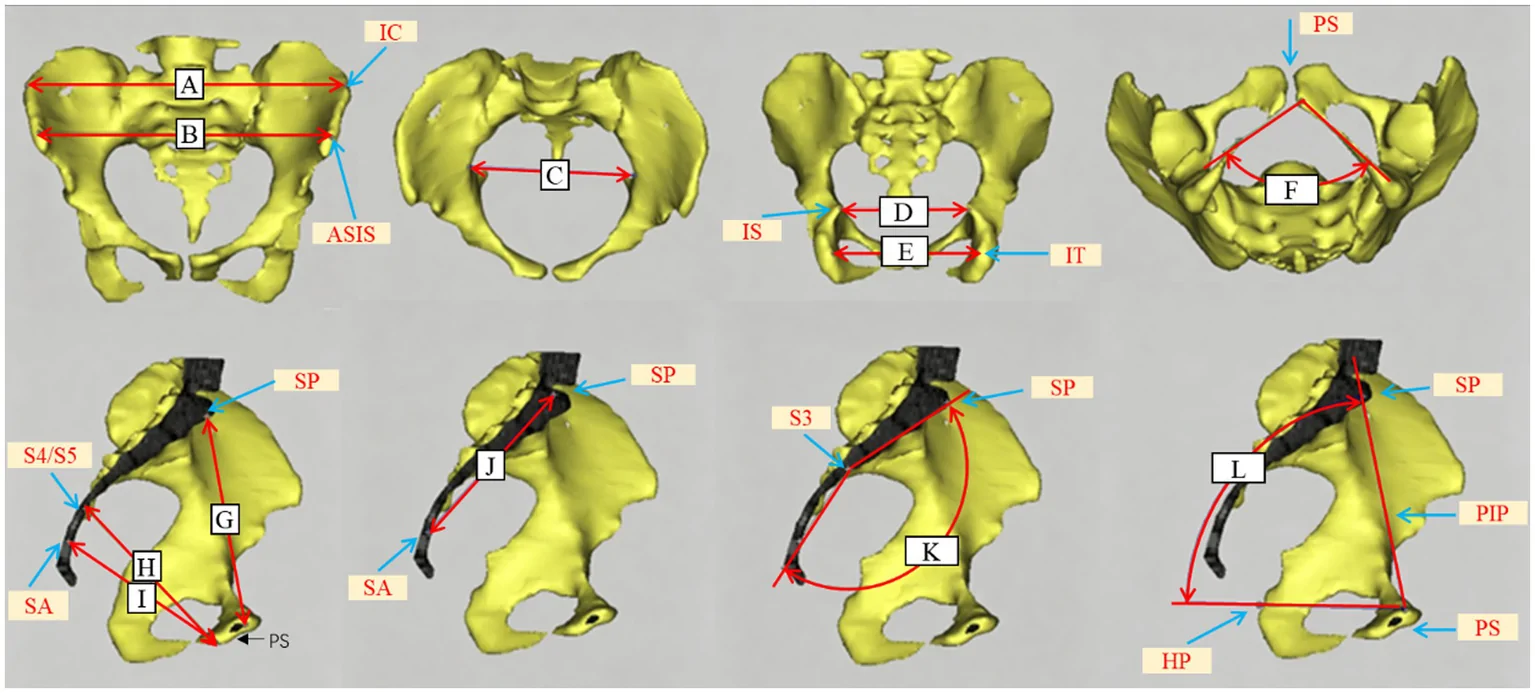
Anatomical landmarks and procedures used for pelvic measurements on MRI-based three-dimensional pelvic models. Red double-headed arrows, lines, and arcs indicate the measured distances or angles, whereas blue arrows indicate the corresponding anatomical landmarks and reference planes. **(A)** Intercristal diameter, measured as the maximum distance between the most lateral points of the bilateral iliac crests. **(B)** External interspinous diameter, measured between the bilateral anterior superior iliac spines. **(C)** Transverse diameter of the pelvic inlet, measured as the maximum transverse distance across the pelvic inlet. **(D)** Transverse diameter of the midpelvis, measured between the bilateral ischial spines. **(E)** Transverse diameter of the pelvic outlet, measured between the inner margins of the bilateral ischial tuberosities. **(F)** Subpubic angle, defined as the angle formed by the bilateral ischiopubic rami. **(G)** Anteroposterior diameter of the pelvic inlet, measured from the sacral promontory to the posterior superior margin of the pubic symphysis. **(H)** Anteroposterior diameter of the midpelvis, measured from the S4–S5 level to the inferior margin of the pubic symphysis. **(I)** Anteroposterior diameter of the pelvic outlet, measured from the sacral apex to the inferior margin of the pubic symphysis. **(J)** Sacral length, measured as the straight-line distance from the sacral promontory to the sacral apex. **(K)** Sacral curvature angle, defined as the angle formed by the sacral promontory, the midpoint of the anterior surface of S3, and the sacral apex, with the S3 point as the vertex. **(L)** Pelvic inclination, defined as the angle between the pelvic inlet plane and the horizontal plane. IC, iliac crest; ASIS, anterior superior iliac spine; IS, ischial spine; IT, ischial tuberosity; PS, pubic symphysis; SP, sacral promontory; SA, sacral apex; S3, third sacral vertebra; S4/S5, S4–S5 level; PIP, pelvic inlet plane; HP, horizontal plane.

A total of 12 pelvic parameters were evaluated: intercristal diameter, external interspinous diameter, transverse and anteroposterior diameters of the pelvic inlet, transverse and anteroposterior diameters of the midpelvis, transverse and anteroposterior diameters of the pelvic outlet, sacral length, sacral curvature angle, subpubic angle, and pelvic inclination. The selection of these parameters and the landmark-based measurement framework were informed by recent CT- and MRI-based pelvimetry studies and by recent research evaluating anatomical landmark identification on three-dimensional pelvic models (10, 20, 29, 30). The exact anatomical landmarks and operational definitions used in the present study are illustrated in Figure 2 and detailed in Supplementary Table 1.

All measurements were independently performed by two trained researchers who were blinded to each other’s measurements. The mean of the two measurements was used for statistical analysis, and the intraclass correlation coefficient was calculated to assess inter-rater reliability.

### 2.7 Clinical pelvic classification

All pelves were classified into gynecoid, platypelloid, anthropoid, and android types according to the Caldwell-Moloy classification system, using the following criteria: Gynecoid: The pelvic inlet is typically transversely oval, with relatively wide iliac wings, generally straight lateral walls, a broad subpubic arch, and relatively non-prominent ischial spines. This is the normal female pelvis, the most common and most favorable for childbirth. Platypelloid: The pelvic inlet is a flattened oval, with the transverse diameter larger than the anteroposterior diameter. The pubic arch is wide; the sacrum loses its normal curvature, becoming straight or deeply curved posteriorly, resulting in a short and shallow pelvis. Anthropoid: The pelvic inlet is a long oval, with the anteroposterior diameter larger than the transverse diameter. The transverse diameters of the inlet, midpelvis, and outlet are all shortened. The lateral pelvic walls converge slightly, and the ischial spines are relatively prominent. The sciatic notch is wide, the pubic arch is narrow, and the sacrum is tilted posteriorly, making the anterior pelvis narrower and the posterior pelvis wider. The sacrum often has six segments and is relatively straight; the anthropoid pelvis is deeper than other types. Android: The pelvic inlet is roughly triangular, with converging lateral walls and prominent ischial spines. The pubic arch is narrow, the sciatic notch is narrow and high-arched, and the sacrum is straight and forward-inclined, with a short posterior sagittal outlet diameter. The pelvic cavity is funnel-shaped, often leading to dystocia. All pelvic classifications were performed jointly by three obstetricians and gynecologists with senior professional titles. If their opinions were inconsistent, the majority opinion determined the pelvic type.

### 2.8 Statistical analysis

Statistical analysis was performed using SPSS 22.0 software. Descriptive statistics, such as mean ± standard deviation (mean ± SD), frequency, and percentage (%), were used to analyze the basic demographic characteristics (Table 1) and pelvic classification data (Table 2). The core data were obtained from measurements of the same participants under two different positions, constituting paired data. The normality of continuous variables was assessed using the Shapiro–Wilk test before statistical comparisons. Therefore, the comparison of means between the two positions (supine vs. hip- and knee-flexed) was performed using the paired-samples t-test to control for individual differences and improve statistical power. The intraclass correlation coefficient (ICC) was used to assess inter-rater reliability. A *p*-value of <0.05 was considered statistically significant.

**Table 2:** Distribution of pelvic clinical types among 27 volunteers.

Type	Gynecoid	Anthropoid	Platypelloid	Android
Number of cases	18	6	3	0
Percentage (%)	66.70	22.20	11.10	0.00

## 3 Results

### 3.1 Baseline characteristics of the study population

A total of 27 nulliparous non-pregnant women of childbearing age who met the inclusion and exclusion criteria were enrolled. As shown in Table 1, the mean age of the sample was 29.5 ± 1.91 years (range 26–32 years), and the mean BMI was 21.94 ± 1.31 kg/m^2^ (range 18.75–24.22 kg/m^2^). All basic demographic characteristics had narrow ranges and small standard deviations (SD), indicating a high degree of homogeneity within the study sample, which provided good baseline control for the subsequent self-paired comparisons.

### 3.2 Three-dimensional pelvic reconstruction and measurement reliability

All 27 volunteers (54 scans in total) successfully underwent pelvic MRI in both the supine and hip- and knee-flexed positions. Based on the optimized MRI sequences, 3D pelvic models were successfully reconstructed in all cases (Figure 1). The image quality was clear, and the anatomical structures were well identified, meeting the requirements for subsequent accurate measurements.

### 3.3 Descriptive statistics of pelvic diameters and angles

The measurement ranges (minimum and maximum values) for all 12 pelvic diameters and angles in the supine and hip- and knee-flexed positions are presented in Table 3.

**Table 3:** Range of pelvic parameters in the supine and hip- and knee-flexed positions.

Measurement parameter	Supine position		Hip- and knee-flexed position	
	Minimum	maximum	Minimum	Maximum
Intercristal diameter (mm)	215.30	293.91	214.01	299.36
External interspinous diameter (mm)	196.52	280.53	196.25	276.16
Transverse diameter of the pelvic inlet (mm)	119.36	147.10	118.78	147.12
Anteroposterior diameter of the pelvic inlet (mm)	113.16	150.66	113.66	147.73
Transverse diameter of the midpelvis (mm)	99.87	139.55	100.38	137.36
Anteroposterior diameter of the midpelvis (mm)	113.55	140.31	111.81	141.08
Transverse diameter of the pelvic outlet (mm)	104.07	153.56	105.63	154.40
Anteroposterior diameter of the pelvic outlet (mm)	107.50	131.49	107.07	133.58
Sacral length (mm)	91.73	122.59	94.34	123.76
Sacral curvature angle (°)	107.11	166.97	131.85	168.03
Subpubic angle (°)	78.46	123.28	77.13	123.40
Pelvic inclination (°)	52.29	79.18	28.70	56.31

### 3.4 Comparison of pelvic parameters between the supine and hip- and knee-flexed positions

**Figure 3:**
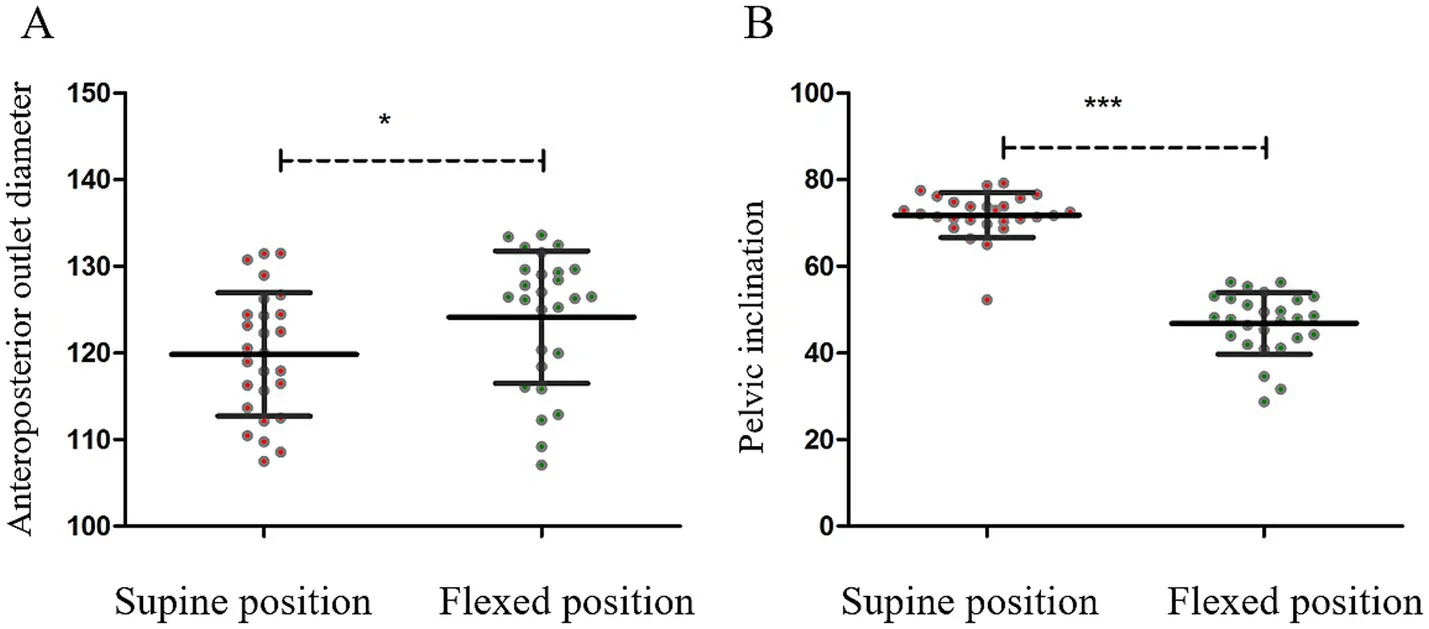
Comparison of key parameters between the supine and hip- and knee-flexed positions. **(A)** Anteroposterior diameter of the pelvic outlet; **(B)** pelvic inclination. *p < 0.05; ***p < 0.001. *p*-values were obtained using paired-samples *t*-tests.

Table 4 presents the paired comparisons of the 12 pelvic parameters between the supine and hip- and knee-flexed positions. Statistically significant positional differences were observed in the anteroposterior diameter of the pelvic outlet and pelvic inclination, whereas no statistically significant differences were detected in the other 10 parameters. As shown in Table 4, the anteroposterior outlet diameter was greater in the hip- and knee-flexed position than in the supine position (124 ± 8 mm vs. 120 ± 7 mm), corresponding to an approximate mean increase of 4 mm (*p* = 0.0361). This increase was visually confirmed in the scatter plot (Figure 3A): In the hip- and knee-flexed position (“flexed”), the vast majority of data points (green) were located above their corresponding supine (“supine”) data points.

**Table 4:** Comparison of three-dimensional pelvic measurements between the supine and hip- and knee-flexed position.

Measurement parameter	Supine position	Hip- and knee-flexed position	*p*
Intercristal diameter (mm)	254 ± 17	253 ± 17	0.8941
External interspinous diameter (mm)	240 ± 19	240 ± 18	0.9993
Transverse diameter of the pelvic inlet (mm)	135 ± 7	135 ± 6	0.8650
Anteroposterior diameter of the pelvic inlet (mm)	133 ± 9	132 ± 8	0.8124
Transverse diameter of the midpelvis (mm)	118 ± 9	118 ± 9	0.9858
Anteroposterior diameter of the midpelvis (mm)	127 ± 7	129 ± 8	0.3652
Transverse diameter of the pelvic outlet (mm)	129 ± 12	130 ± 11	0.9085
Anteroposterior diameter of the pelvic outlet (mm)	120 ± 7	124 ± 8	0.0361
Sacral length (mm)	112 ± 7	113 ± 7	0.6786
Sacral curvature angle (°)	146 ± 14	149 ± 11	0.3563
Subpubic angle (°)	95 ± 11	95 ± 11	0.8948
Pelvic inclination (°)	72 ± 5	47 ± 7	0.0001

Data are presented as mean ± standard deviation. *p*-values were obtained using paired-samples *t*-tests. A two-sided *p*-value <0.05 was considered statistically significant.

Significantly Altered Pelvic Axial Alignment: The second key finding was a significant change in pelvic inclination. Pelvic inclination was lower in the hip- and knee-flexed position than in the supine position (47 ± 7° vs. 72 ± 5°), corresponding to an approximate mean reduction of 25° (*p* < 0.001). Figure 3B shows the data distribution under the two positions: The data points for the hip-knee flexed position (“flexed”) formed a cluster completely separated from those of the supine position (“supine”) at lower angles, indicating a consistent and pronounced positional difference within the present sample.

Other Pelvic Parameters Without Statistically Significant Positional Differences: Notably, the other 10 pelvic parameters showed no statistically significant differences between the two positions (all *p* > 0.05). As shown in Table 4, parameters without statistically significant positional differences included all relevant diameters of the pelvic inlet (transverse inlet diameter and anteroposterior inlet diameter), midpelvis (transverse midpelvic diameter and anteroposterior midpelvic diameter), and external pelvic (intercristal diameter and external interspinous diameter) measurements. These findings should be interpreted cautiously because the limited sample size may have reduced the ability to detect small positional differences in the other parameters. Detailed scatter plots of the negative results are provided in Supplementary Figure 1.

### 3.5 Clinical pelvic classification

**Figure 4:**
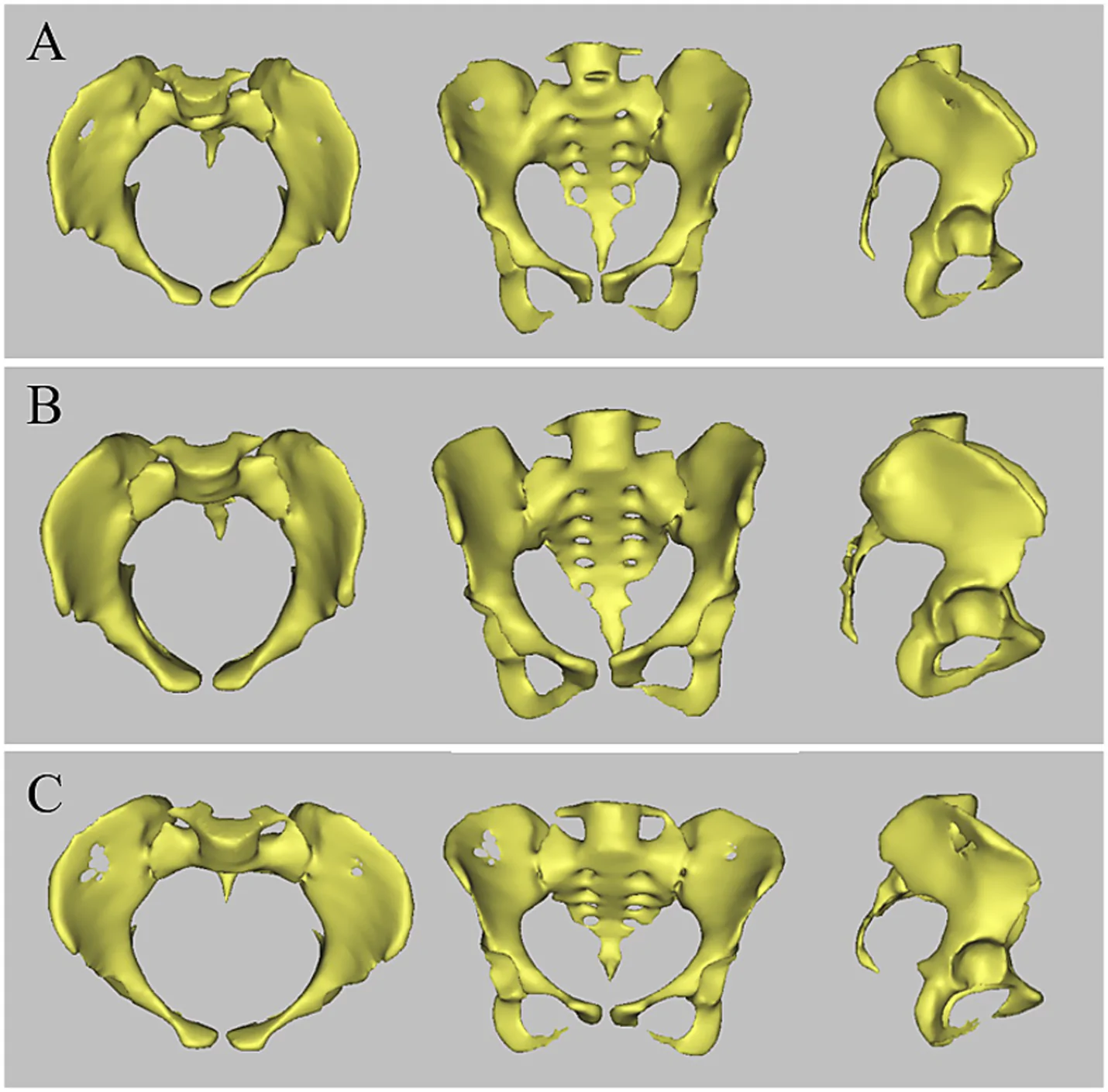
Representative models of clinical pelvic classification: **(A)** Gynecoid type; **(B)** Anthropoid type; **(C)** Platypelloid type.

The results of clinical pelvic classification for the 27 volunteers are shown in Table 2. The gynecoid pelvis was the most common type, observed in 18 cases (66.70%), followed by the anthropoid pelvis in six cases (22.20%), and the platypelloid pelvis in three cases (11.10%). No android pelvis was identified in this sample (0%). Three-dimensional models of the three representative pelvic types (gynecoid, anthropoid, and platypelloid) are shown in Figure 4.

## 4 Discussion

In this study, using an optimized, radiation-free MRI-based three-dimensional (3D) reconstruction technique, we quantitatively compared *in vivo* pelvic morphological changes in nulliparous women between the supine position and the hip- and knee-flexed position. The results showed that this postural change was primarily associated with alterations in the pelvic outlet and pelvic axial alignment.

The following two key findings emerged from this study: (1) compared with the supine position, the hip- and knee-flexed position significantly increased the anteroposterior outlet diameter (mean increase 4 mm, *p* = 0.0361) and (2) the hip- and knee-flexed position significantly decreased pelvic inclination (mean reduction 25°, *p* < 0.001). No statistically significant differences were observed in the measured diameters of the pelvic inlet, midpelvis, or external pelvic measurements (all *p* > 0.05). These findings have potential biomechanical relevance but should not be interpreted as direct evidence of improved delivery outcomes. The observed increase in the anteroposterior outlet diameter may partially reflect the biomechanical changes associated with the McRoberts maneuver. Previous radiographic and biomechanical studies have shown that the maneuver produces the cephalad rotation of the pubic symphysis, flattening of the sacrum, and reduction of lumbar lordosis (27, 28). However, because sacroiliac joint motion, pubic symphysis displacement, fetal descent, and clinical delivery outcomes were not directly measured, the present study cannot establish the evidence that outlet enlargement is the principal mechanism by which the McRoberts maneuver resolves shoulder dystocia. Accordingly, the hip- and knee-flexed position evaluated in the present study should be regarded as a posture intended to approximate the principal hip-flexion component of the McRoberts maneuver rather than as an exact reproduction of every element of the clinical maneuver. Therefore, the observed pelvic changes should be attributed specifically to the position examined in this study and should not be generalized to the complete clinical McRoberts maneuver. Similarly, the absence of statistically significant changes in inlet and midpelvic dimensions should not be interpreted as proof that this position acts exclusively on outlet obstruction.

The mean increase of 4 mm in the anteroposterior outlet diameter observed in this study, although statistically significant (*p* = 0.0361), was modest in magnitude. Therefore, this finding should be interpreted as evidence of a measurable positional change in pelvic morphology rather than as direct evidence of a clinically meaningful improvement in the birth canal.

Furthermore, the increase observed in our study was smaller than that reported for some weight-bearing delivery positions, such as squatting. Hemmerich et al. (8) reported in a computational simulation that the final squatting posture increased the anteroposterior and transverse pelvic outlet diameters by approximately 6.1 and 11.0 mm, respectively, under pregnant simulation conditions. This quantitative difference may reflect the different biomechanical characteristics of the positions assessed. The McRoberts maneuver involves marked hip flexion and may modify pelvic orientation through rotation of the pubic symphysis and sacrum (28), whereas squatting and other upright positions additionally involve weight bearing and gravity, which may produce different patterns and magnitudes of pelvic change (6). Nevertheless, because joint motion and loading were not directly measured in the present study, the mechanisms underlying the observed differences remain uncertain. Whether a 4-mm increase in outlet diameter contributes to clinically meaningful benefits during actual labor requires further investigation in pregnant women and correlation with delivery outcomes. Previous imaging and modeling studies provide important context for interpreting the modest 4-mm increase observed in the present study. Michel et al. (5) and Reitter et al. (6) demonstrated that upright or kneeling squat positions alter selected midpelvic and outlet dimensions on MRI. Among pregnant women at gestational week 32, Kjeldsen et al. (7) reported that the anteroposterior pelvic outlet was 2 mm larger in the semi-lithotomy position and 9 mm larger in the kneeling squat position than in the supine position. Differences in the magnitude and direction of positional changes among these studies may reflect pregnancy status, weight-bearing conditions, the degree of hip and knee flexion, and differences in imaging, modeling, and anatomical landmark definitions. In addition, the McRoberts maneuver differs biomechanically from weight-bearing or kneeling squat positions. Therefore, the 4-mm increase observed in the present study should be viewed as complementary to previous position-related imaging findings rather than as directly comparable with studies of squatting. Its clinical significance during actual labor remains uncertain (9). Accordingly, the present study was not designed to identify the optimal birth position or compare clinical delivery positions but rather to characterize the pelvic morphological effect of a specific controlled hip-flexion posture.

Clinical evidence regarding the association between maternal position and birth outcomes remains heterogeneous. Systematic-review evidence suggests that upright positions may be associated with shorter labor duration and lower rates of certain obstetric interventions in some populations; however, the magnitude and direction of effects vary according to the stage of labor, epidural analgesia status, the specific position adopted, and the outcome assessed (1, 2). The certainty of evidence has ranged from very low to moderate, and the overall benefits and risks of individual positions have not been conclusively established (2). Moreover, late-pregnancy MRI findings have not yet provided reliable predictors of labor outcomes (9). Accordingly, the morphological changes observed in the present study may offer a plausible anatomical basis for maternal repositioning but cannot demonstrate that the hip- and knee-flexed position improves vaginal delivery outcomes.

The strengths of this study include the following. First, MRI-based 3D reconstruction enabled radiation-free *in vivo* measurement of the bony pelvis. Although CT-based 3D reconstruction has been widely used for accurate pelvimetry (13–15), its use in obstetric and volunteer research is limited by ionizing radiation. Conventional MRI has historically been limited by suboptimal visualization of cortical bone (24). However, previous comparative research has demonstrated good agreement and reproducibility between MRI-based and CT-based 3D pelvic models, supporting the reliability of MRI-based 3D pelvimetry (22). Using optimized MRI sequences combined with Mimics software, the present study obtained measurable 3D models without exposing participants to ionizing radiation. Second, the paired design allowed each participant to serve as her own control, thereby reducing the influence of fixed inter-individual differences in baseline pelvic morphology. Third, all measurements were independently performed by two trained raters who were blinded to each other’s measurements. The inter-rater ICCs were reported to be >0.90, suggesting high measurement reproducibility.

This study also has limitations. (1) Sample size (*N* = 27): First, the sample size was relatively small. Although the prospective paired design allowed each participant to serve as her own control and reduced the influence of inter-individual variation in pelvic morphology, the limited sample size may have reduced the ability to detect subtle positional changes, particularly for parameters with small effect sizes. Therefore, the present findings should be considered preliminary biomechanical observations requiring validation in larger and more diverse populations. (2) Limited ethnic generalizability—All participants were Asian nulliparous women recruited from a single center in China and were within prespecified ranges of age, height, weight, and BMI. Because pelvic morphology may vary across ethnic populations, the generalizability of these findings to other Asian populations, non-Asian populations, and women with different body habitus may be limited.

(3) Non-pregnant status and unmeasured hormonal factors—All participants were non-pregnant, and the menstrual-cycle phase, reproductive hormone concentrations, and hormonal contraceptive use were not recorded. A recent systematic review and meta-analysis indicated that reproductive hormones, particularly estradiol and relaxin, may influence the mechanical and cellular properties of several ligaments, including intrapubic ligaments. However, current evidence suggests that menstrual-cycle-related variations are unlikely to produce clinically meaningful changes in ligament laxity, and direct evidence regarding menstrual-cycle-related changes in adult pelvic mobility remains limited (31). Pregnancy-related pelvic girdle disorders also have complex etiological and pathophysiological characteristics rather than being attributable to a single hormonal factor (32). Therefore, the specific influence of menstrual-cycle phase or reproductive hormone status on the positional changes observed in this study remains uncertain. Because these variables were not assessed, residual hormonal variability should be acknowledged when interpreting the modest 4-mm increase in the anteroposterior outlet diameter. The present study cannot determine whether pregnancy-related physiological changes would amplify, attenuate, or otherwise modify the observed positional effect. Future studies should prospectively record menstrual-cycle phase, hormonal contraceptive use, and, where feasible, reproductive hormone concentrations.

Based on the findings and limitations of this study, future research should (1) conduct large, multicenter studies with prospectively defined primary outcomes and sample-size calculations based on expected within-participant changes; (2) conduct further MRI-based 3D reconstruction studies in non-pregnant women while prospectively recording the menstrual cycle phase, hormonal contraceptive use, and relevant reproductive hormone concentrations and perform complementary studies in pregnant women while recording gestational age and relevant obstetric characteristics; and (3) link imaging findings to actual delivery outcomes, including labor duration, fetal descent, shoulder dystocia, operative vaginal delivery, and cesarean delivery, to determine whether position-related morphological changes translate into clinical benefit.

## 5 Conclusion

This study provides quantitative, radiation-free imaging evidence of pelvic morphological changes associated with a hip- and knee-flexed position in nulliparous non-pregnant women. This position was associated with a modest increase in the anteroposterior outlet diameter and a marked reduction in pelvic inclination. These findings provide preliminary evidence of position-related pelvic mobility and may contribute to understanding the biomechanical basis of the McRoberts maneuver. However, they should not be interpreted as direct evidence that the position improves fetal descent, resolves outlet obstruction, or increases the likelihood of vaginal delivery. Further validation in larger pregnant populations and correlation with actual delivery outcomes are required.

## Data Availability

The original contributions presented in the study are included in the article/Supplementary material; further inquiries can be directed to the corresponding author.

## Supplementary material

SUPPLEMENTARY FIGURE 1 Scatter plots of three-dimensional pelvic parameter measurements obtained in the supine position versus the hip-knee flexed position. **(A)** External interspinous diameter; **(B)** anteroposterior diameter of the pelvic inlet; **(C)** transverse diameter of the pelvic inlet; **(D)** transverse diameter of the midpelvis; **(E)** anteroposterior diameter of the midpelvis; **(F)** transverse diameter of the pelvic outlet; **(G)** subpubic angle; **(H)** sacral length; and **(I)** sacral curvature angle. ns, not statistically significant (*p* ≥ 0.05). *p* values were obtained using paired-samples *t*-tests.
