# Supplementary material for: Impact of supine vs. hip- and knee-flexed position on anteroposterior diameter and inclination of the female pelvic outlet: an MRI-based 3D reconstruction study
Source: Front Med (Lausanne). 2026 Aug 21;13:1895286. doi: 10.3389/fmed.2026.1895286 (PMC13541679; doi:10.3389/fmed.2026.1895286)
Supplement: Supplementary file 1 [file Table_1.docx]

| **Supplementary Table 1. Anatomical landmarks and operational definitions of pelvic measurements** | | | | |
| --- | --- | --- | --- | --- |
| **Code** | **Measurement parameter** | **View** | **Anatomical landmarks and measurement procedure** | **Unit** |
| A | Intercristal diameter | Anterior | Maximum distance between the most lateral points of the bilateral iliac crests | mm |
| B | External interspinous diameter | Anterior | Distance between the bilateral anterior superior iliac spines | mm |
| C | Transverse diameter of the pelvic inlet | Superior | Maximum transverse distance across the pelvic inlet | mm |
| D | Transverse diameter of the midpelvis | Posterior | Distance between the bilateral ischial spines | mm |
| E | Transverse diameter of the pelvic outlet | Posterior | Distance between the inner margins of the bilateral ischial tuberosities | mm |
| F | Subpubic angle | Inferior/anterior | Angle formed by the bilateral ischiopubic rami | ° |
| G | Anteroposterior diameter of the pelvic inlet | Midsagittal | Distance from the sacral promontory to the posterior superior margin of the pubic symphysis | mm |
| H | Anteroposterior diameter of the midpelvis | Midsagittal | Distance from the S4–S5 level to the inferior margin of the pubic symphysis | mm |
| I | Anteroposterior diameter of the pelvic outlet | Midsagittal | Distance from the sacral apex to the inferior margin of the pubic symphysis | mm |
| J | Sacral length | Midsagittal | Straight-line distance from the sacral promontory to the sacral apex | mm |
| K | Sacral curvature angle | Midsagittal | Angle formed by the sacral promontory, the midpoint of the anterior surface of S3, and the sacral apex, with S3 as the vertex | ° |
| L | Pelvic inclination | Midsagittal | Angle between the pelvic inlet plane and the horizontal plane | ° |
| **Notes:** Codes A–L correspond to the measurement labels shown in Figure 2. Linear measurements are reported in millimeters, and angular measurements are reported in degrees. The external interspinous diameter refers to the distance between the bilateral anterior superior iliac spines and is distinct from the transverse diameter of the midpelvis, which is measured between the bilateral ischial spines. The selection of parameters and the landmark-based measurement framework was informed by previous CT- and MRI-based pelvimetry studies. | | | | |
